# The Role of the P1 Latency in Auditory and Speech Performance Evaluation in Cochlear Implanted Children

**DOI:** 10.1155/2022/6894794

**Published:** 2022-04-05

**Authors:** Shan Xiong, Liwei Jiang, Yu Wang, Tao Pan, Furong Ma

**Affiliations:** ^1^Department of Otolaryngology Head and Neck Surgery, Peking University Third Hospital, Beijing 100191, China; ^2^Department of Otolaryngology Head and Neck Surgery, The Affiliated Hospital of Qingdao University, Qingdao, Shandong Province, 266000, China

## Abstract

Auditory deprivation affects normal age-related changes in the central auditory maturation. Cochlear implants (CIs) have already become the best treatment strategy for severe to profound hearing impairment. However, it is still hard to evaluate the speech-language outcomes of the pediatric CI recipients because of hearing-impaired children with limited speech-language abilities. The cortical auditory evoked potential (CAEP) provides a window into the development of the auditory cortical pathways. This preliminary study is aimed at assessing electrophysical characteristics of P1-N1 of electrically CAEP in children with CIs and at exploring whether these changes could be accounted for in auditory and speech outcomes of these patients. CAEP responses were recorded in 48 children with CIs in response to electrical stimulus to determine the presence of the P1-N1 response. Speech perception and speech intelligibility of the implanted children were further evaluated with the categories of auditory performance (CAP) test and speech intelligibility rating (SIR) test, respectively, to explore the relationship between the latency of P1-N1 and auditory and speech performance. This study found that P1 and N1 of the intracochlear CAEP were reliably evoked in children fitted with CIs and that the latency of the P1 as opposed to that of N1 was negative in relation to the wearing time of the cochlear implant. Moreover, the latency of the P1 produced significantly negative scores in both CAP and SIR tests, which indicates that P1 latency may be reflective of the auditory performance and speech intelligibility of pediatric CI recipients. These results suggest that the latency of P1 could be used for the objective assessment of auditory and speech function evaluation in cochlear-implanted children, which would be helpful in clinical decision-making regarding intervention for young hearing-impaired children.

## 1. Introduction

Cochlear implantation (CI) has become a widely accepted intervention in the treatment of severe to profound deafness in children with normal cognition since 1980 [1]. Epidemiological studies estimate that as many as 40% of hearing-impaired children have an additional need for CI [2]. Cochlear implantation has a positive impact on the quality of life in deaf children, which not only bring improved social-emotional abilities to the profoundly deaf children but also help improve attention span and adherence to other therapies and school activities [3, 4]. However, it is still a challenge to evaluate postintervention rehabilitation for pediatric hearing-impaired patients receiving cochlear implant. Clinical assessment after cochlear implantation is usually based on speech perception tests. For children of whom behavioral audiometric responses may be unreliable (e.g., infants and children with multiple disabilities), objective measures are needed to evaluate the CI performance in infants and young children with profound deafness.

The auditory brainstem response (ABR) is used for the measurement for CI performance. The cortical auditory evoked potential (CAEP) is an objective measure of human speech encoding in individuals with normal or impaired auditory systems. It allows for reliable responses at higher levels of the brain than ABR can provide [5] and be used to assess the outcomes of cochlear implants in children [6]. Exogenous components (P1 and N1) are the main components of the CAEP response and can provide information on cortical auditory processing [7, 8]. According to a previous study [9–11], the P1 response, a positive peak, is reliably evoked in school age children after the auditory stimulus. P1 peak latency is around 85–95 ms and becomes shorter with increasing age, declining to an adult value around 40–60 ms. Following P1, a negative peak N1 occurs with its latency around 100–150 ms in 5–6 years old and with increasing age; the N1 becomes the most prominent of peaks, which occurs at a poststimulus latency around 100 ms.

The age-dependent shorter latency of P1 is thought to be reflective of more efficient synaptic transfers and more efficient auditory pathways [12]. The P1 is also suggested to reflect functional synaptic activity in the central auditory system, providing information regarding auditory cortical maturation [13]. Due to its good temporal resolution, P1 has been regarded as a noninvasive indicator of central auditory pathway maturation in pediatric hearing loss children with cochlear implants [14, 15].

A study by Rich et al. [16] found that when compared to age-matched peers with normal hearing, children with hearing loss wearing CIs exhibited less mature social skills with fewer quality friendships. Several studies have evaluated the hearing and speech outcomes following cochlear implantation in children using the categories of auditory performance (CAP) and speech intelligibility rating (SIR) [17]. However, those tests are difficult to be measured in young children. Moreover, speech and language development in children fitted with cochlear implants is related to the maturation of the central auditory system [9]. The disorders at the higher levels of the auditory pathway may be also contributed to the unsuccess of the cochlear implant in children.

A few case studies reported in the literature have shown that CAEP are of great value in providing objective data on the functionality of auditory cortical structures and maturity of the central auditory system [15, 18]. Whether CAEP could also be a biomarker for assessing the auditory and speech functions in hearing-impaired children with CIs is a question that remained to be explored. In 1990, Shannon et al. [19] designed a computer interface that allowed the presentation of biphasic pulse stimuli in patients with the nucleus cochlear implant, which made it possible to detect electrical stimulation CAEP in CI patients. Accordingly, this study was designed to address the issue of whether the electrical stimulation CAEP measure (P1 and N1) could be regarded as a potential objective indictor for assessing the auditory performance and speech intelligence of deaf children fitted with cochlear implants. In this study, the intracochlear CAEPs were performed, and CAP and SIR were applied to measure the auditory performance and speech intelligibility outcomes in pediatric CI recipients to investigate the electrophysical characteristics of the electrically evoked CAEPs and the relationship between the components of CAEP, mainly the P1 and N1, and the auditory performance and speech intelligibility outcomes.

## 2. Material and Method

### 2.1. Participants

Research protocols were approved by the ethics committees of Peking University Third Hospital. Parental consent was also obtained for all children included in the study. 48 children with prelingually profound deafness that received cochlear implants in accordance to the surgical criteria for cochlear implants in our department by the same surgeon and received rehabilitation at Beijing Bao Di Kang Company from 2008 to 2015 were selected to our clinical study. Electrically, P1-N1 cortical auditory evoked potential (CAEP) was tested in 48 subjects. The subjects were required to be alert during the test by watching cartoon movies. Among them, 5 subjects were excluded from the study because they did not complete the CAEP test. The remaining 43 subjects (30 male, 13 females; age ranged from 2.09 to 11.00 years) successfully finished the CAEP. For the 43 patients, 24 subjects unilaterally received cochlear implants in their right ears and 19 in their left ears. Of the 43 participants, 35 were implanted with the CI24RE Nucleus Freedom™ implant (Cochlear Ltd., Sydney, New South Wales, Australia), and 8 were implanted with the Cochlear Nucleus CI512 implant (Cochlear Ltd., Sydney, New South Wales, Australia). Detailed descriptions of 43 subjects in this study are provided in Table 1.

### 2.2. Intracochlear CAEP Recording

We measured the responses and latencies of P1 and N1 of the CAEP in response to electrical stimuli; the CAEP recording was performed with two computers installed with the stimulation system Custom Sound™ EP 4.0 software from Cochlea™ and the recording system, Bio-logic® AEP Version 7.0.0 software, respectively. The stimulus was bipolar alternating mode with a 10 CL (current level) step of stimulation intensity. The stimulus current was a 200 ms × 10 sequential electrical stimulation with 2 ms intervals within the sequence, and the stimulus duration was 20 ms at a rate of 1.1 times/s. During the CAEP test, electrically intracochlear stimuli was performed by the Electrode 20, while Electrode 10 was used as the reference electrode. Responses were recorded with the Bio-logic Navigator Pro AEP (auditory evoked potentials) system (Natus Medical, Inc., Pleasanton, CA) triggered by the stimulus output of the programming interface. The recording electrode was placed on the middle of the child's forehead. The common reference and the reference electrode were placed between the eyebrows and at the contralateral mastoid processes, respectively. All electrode impedances were less than 5 k*Ω* and connected to an RF-shielded amplifier with a gain of 100,000.

The stimulation and recording parameters selected were based on the previous research on intracochlear CAEP [20–22]. During the CAEP recording, the children were allowed to watch animation to keep calm and focused in a quiet room.

After 1-70 Hz second digital filtering, the waveforms of the CAEP were judged according to the literatures [22]. CAEP P1 latency and N1 latency were obtained and analyzed.

### 2.3. Evaluation of Auditory Performance and Speech Intelligibility Outcomes

Among the 48 subjects, 43 subjects elicited the typical P1-N1 CAEP and were further evaluated for auditory performance and speech intelligibility after cochlear implantation by categories of auditory performance (CAP) and intelligibility rating scale (SIR) scores. The CAP scale, which is an index consisting of eight performance categories arranged in order of increasing difficulty [23], was employed to assess the speech performance of the postimplanted children. For evaluating the speech intelligibility of deaf children after cochlear implantation, SIR was also obtained, which is a reliable rating scale in a format that is understood by parents, local professionals, and health care purchasers [24]. All the tests were conducted in a quiet room by the same audiological team in our department via face-to-face interviews with the parents of the 43 children in this study, according to our previous study [25].

### 2.4. Statistical Analysis

Electrophysiological characteristics of P1-N1 electrically CAEP were described and analyzed. Statistical analysis was performed using the SPSS 22.0 software. The latency values of the P1-N1 of CAEP were presented as the mean ± standard deviation (SD). Pearson's chi-square test was performed for the detection rate of P1 and N1 according to implantation side and gender. An independent samples *t*-test was used to compare the mean latency values of the P1 and N1 obtained in this study from the implantation side and gender. The Spearman rank correlation analysis was also employed to analyze (1) the interactions between the latency values of the P1 and N1 during age of implantation, the time of evaluation postimplantation, and the wearing time of cochlear implant, (2) the relations between the scores of CAP and SIR and age at implantation, the time of evaluation postimplantation, and the wearing time of cochlear implant, and (3) the correlations between the CAEP measures (latency of the P1 and latency of the N1) and auditory and speech performance as measured by CAP growth and SIR growth. Statistical significance was considered with a *P* value less than 0.05.

## 3. Result

### 3.1. Characteristics of P1-N1 CAEP Waveform

**Figure 1:**
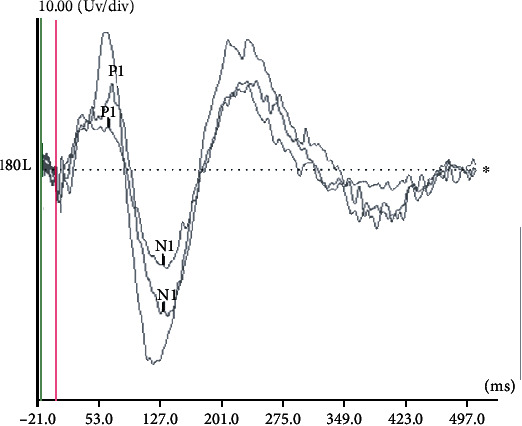
Typical CAEP waveform of the same subject at different levels of stimulation different electrical intensities.

**Figure 2:**
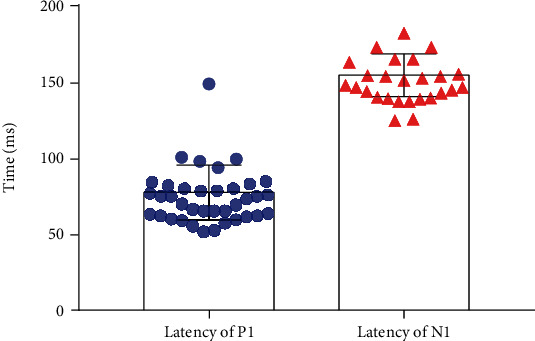
The latency values of the P1 and N1 of electrically evoked cortical auditory evoked potential (P1 waves (*n* = 37) and N1 waves (*n* = 26)).

Of the 43 children who completed the CAEP test, 37 subjects reliably elicited the typical P1-N1 CAEP waveform, and the evoking rate of electrically evoked CAEP was 86.0%.  Figure 1 displays the typical electrically evoked CAEP from a subject at different stimulation intensities. Among the 43 subjects, the P1 and N1 were presented in the 86.0% of the children (*n* = 37) and 60.5% of the children (*n* = 26). The P2 latency value was 77.71 ± 17.97 ms, and the N2 latency value was 154.84 ± 14.12 ms (Figure 2).

The chi-square test was conducted on the detection rates of P1 and N1 waves found in the implantation side and gender, and it was found that the detection rates of P1 and N1 had no correlation with the implantation side and gender (*P* > 0.05), as well as the relationship between the latency values of the P1 and N1 waves and the implantation side and gender, as shown in Tables 2 and 3, respectively.

### 3.2. Relationship between the Latency Values of the P1 and N1 and the Age of Implantation, the Time of Evaluation Postimplantation, and Wearing Time of the Cochlear Implant

**Figure 3:**
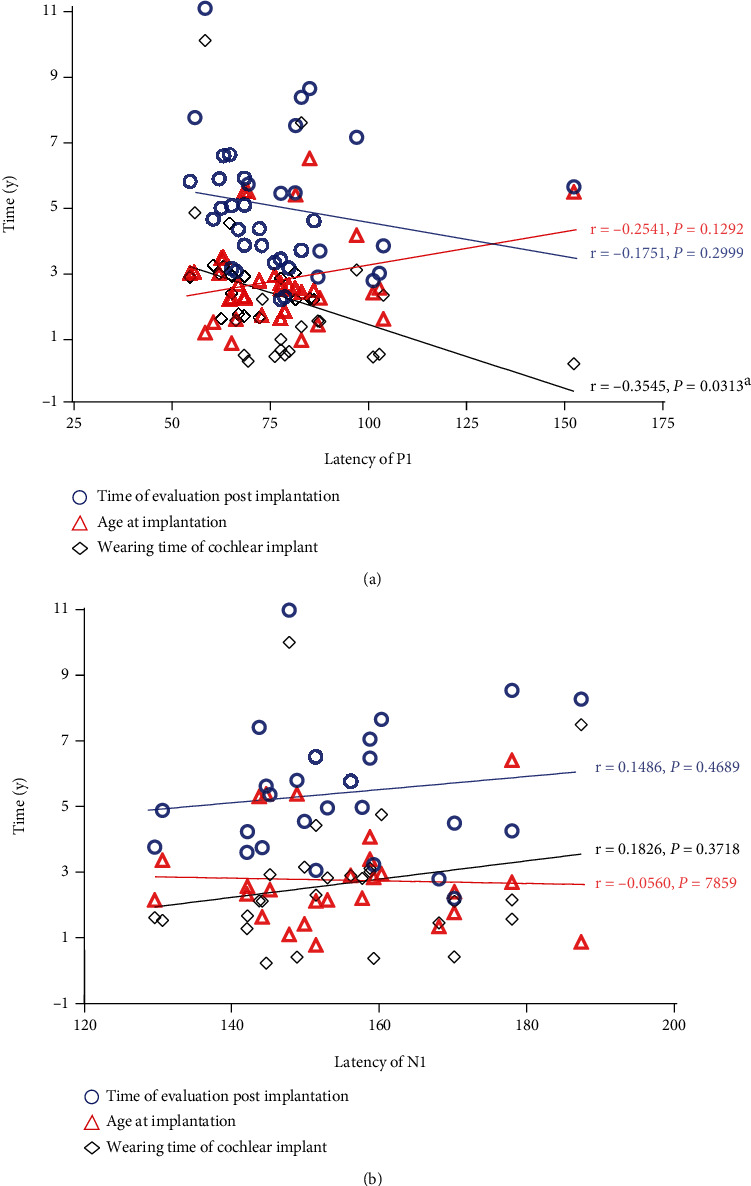
(a) The correlations of the latency of P1 measured in children during the age of implantation, the time of evaluation postimplantation, and wearing time of the cochlear implant. (b) The correlations of the latency of N1 measured in children with the age of implantation, the time of evaluation postimplantation and wearing time of cochlear implant. Blue hollow circles represent the time of evaluation postimplantation, the red triangles represent age at implantation, and the black rhombuses represents wearing time of the cochlear implant (^a^Statistical significance, *P* < 0.05).

The results of the Spearman correlation tests illustrated by the scatterplot between the latency values of P1/N1, the age of implantation, the time of evaluation postimplantation, and the wearing time of cochlear implant are listed in Figure 3. No significant correlations were found between the latency of P1 and the age of implantation (*r* = −0.2541, *P* = 0.1292), as well as between the latency of P1 and the time of evaluation postimplantation (*r* = −0.1751, *P* = 0.2999). The latency of P1 was found to have a significantly negative association with the wearing time of cochlear implant (*r* = −0.3545, *P* = 0.0313). In terms of N1 latency, there is no significant correlation between the age at implantation (*r* = −0.0560, *P* = 0.7859), the time of evaluation postimplantation (*r* = 0.1486, *P* = 0.4689), and the wearing time of the cochlear implant (*r* = 0.1826, *P* = 0.3718).

### 3.3. Relationship between the Scores of the CAP and SIR Test and the Age of Implantation, the Time of Evaluation Postimplantation, and the Wearing Time of Cochlear Implant

**Figure 4:**
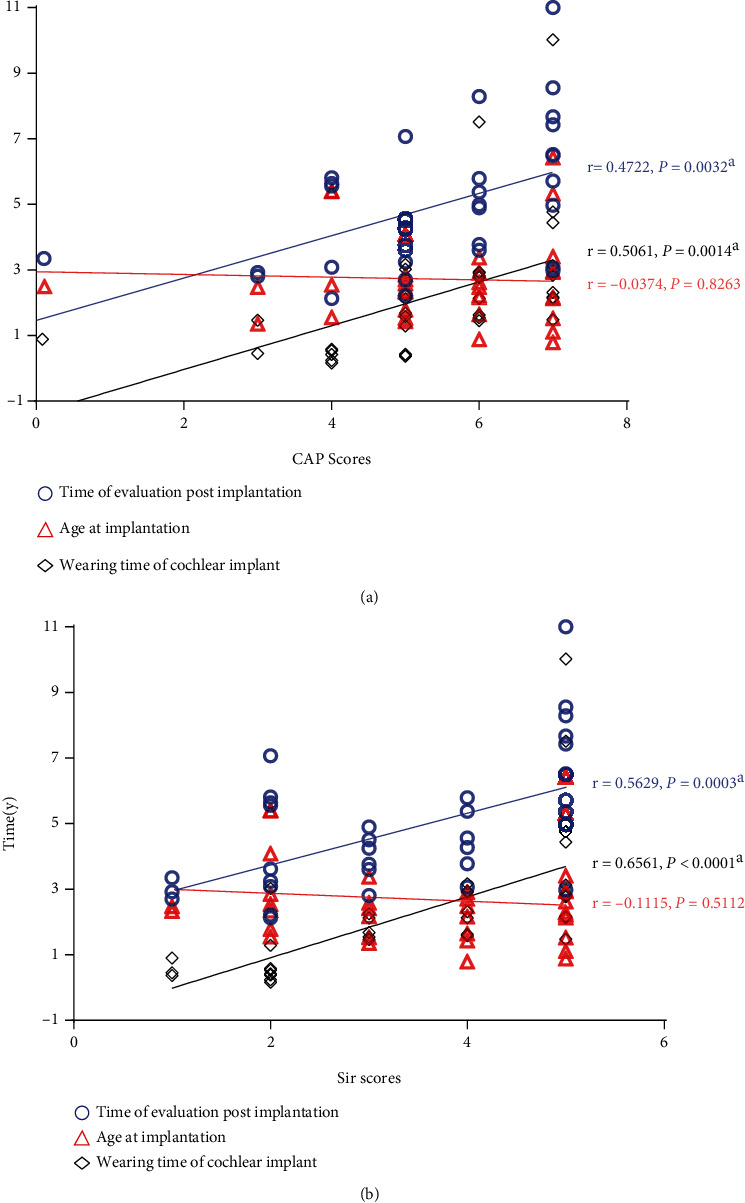
(a) The correlations of the CAP scores measured in children during the age of implantation, the time of evaluation postimplantation, and wearing time of the cochlear implant. (b) The correlations of the SIR Scores measured in children with the age of implantation, the time of evaluation postimplantation, and wearing time of the cochlear implant. The blue hollow circle represents the time of evaluation postimplantation, the red triangle represents age at implantation, and the black rhombus represents wearing time of the cochlear implant (^a^Statistical significance, *P* < 0.05).

The categories of auditory performance (CAP) and speech intelligibility rating (SIR) were employed to evaluate the auditory performance and speech intelligibility in our subjects after cochlear implant. The scores of the CAP and SIR results were compared with wearing time of the cochlear implant, the time of evaluation postimplantation, and the age at implantation. As the scatterplot shown in Figure 4, the results revealed that the values of both tests were positively correlated with wearing time of the cochlear implant (*r* = 0.5061, *P* = 0.0014 (CAP) versus *r* = 0.6561, *P* < 0.0001 (SIR)) and with the time of evaluation postimplantation (*r* = 0.4722, *P* = 0.0032 (CAP) versus *r* = 0.5629, *P* = 0.0003 (SIR)). However, no correlation was found between the results of the CAP and SIR scales and the age at implantation (*r* = −0.0374, *P* = 0.8263 (CAP) versus *r* = −0.1115, *P* = 0.5112 (SIR)).

### 3.4. Relationship between the Latency Values of P1 and N1 and Auditory Performance and Speech Intelligibility

**Figure 5:**
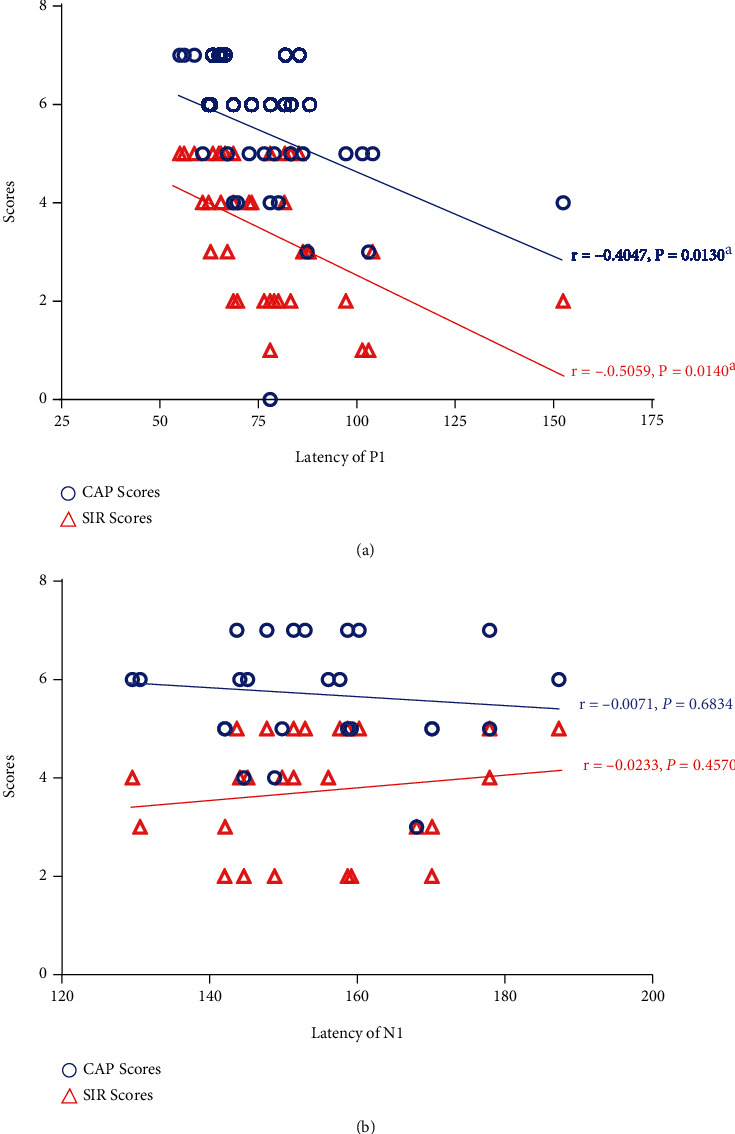
(a) The correlations of the latency of P1 measured in children with the CAP scores and the SIR scores. (b) The correlations of the latency of N1 measured in children with the CAP scores and the SIR scores. The blue hollow circles represent the CAP scores, and the red triangles represent the SIR scores (^a^Statistical significance, *P* < 0.05).

To determine the relationship between the latency values of P1 and N1 and auditory performance and speech function in children receiving cochlear implants, we further compared the CAP and the SIR results with the latency values of P1 and N1. As shown in Figure 5, scores of CAP and SIR have significantly negative correlations with the latency value of the P1 (*r* = −0.4047, *P* = 0.0130 (CAP) and *r* = −0.5059, *P* = 0.0140 (SIR), respectively). However, there was no significant correlation between N1 latency and the scores of CAP and SIR (*r* = 0.0071, *P* = 0.6834 (CAP) and *r* = 0.0233, *P* = 0.4570 (SIR), respectively). These results suggested that the latency of the CAEP peak P1 is related to the auditory and speech performance evaluated by the scores of the CAP and the SIR in pediatric CI recipients.

## 4. Discussion

Assessment through surgery and postoperative rehabilitation is required for children receiving cochlear implants, and it is difficult to predict what level of benefit these children may derive from cochlear implantation. The rapid alterations to morphology and decreases in the latency of CAEP within the first 8 months following implantation indicated a high degree of plasticity in the central auditory pathways of congenitally deaf children after early cochlear implantation [15]. The early-implant children show higher levels of language scores and better speech perception outcomes, which suggests the development of the central auditory system of congenitally deaf children [26, 27]. The relationship between the CAEP and the speech function in children with cochlear implants remains unknown.

P1 CAEP recorded in response to a synthesized speech syllable /ba/ was present in all children with auditory neuropathy spectrum disorder after implantation [28]. Rance et al. [29] found that CAEPs for tones and speech tokens were present in over 85% of those with sensorineural hearing loss (SNHL) but for only 60% of those with auditory neuropathy spectrum disorder (ANSD). However, in this study, only 86% of the deaf children who used cochlear implants show CAEP responses after electrically stimulation.

Sharma et al. [30] found that the feature of CAEP in normal children ranging in age from 6 to 15 years elicited by synthesized consonant-vowel syllable (ba) is a P1 response at about 100 ms and a negative wave named N1 at about 200 ms. The electrically stimulation CAEP recorded in this study is characterized by a large P1 response at about 77.71 ms, followed by a broad negativity N1 response at about 154.84 ms. The latency of P1 and N1 components of the CAEP response in our study is shorter compared with the Sharma et al. finding; the reason may be that the stimulus is different.

In this study, the latency of the P1 and the latency of the N1 of the electrically CAEP were not related to the age of implantation, nor were they related with the time of evaluation postimplantation. There was a significantly negative correlation between the P1 latency of the electrical CAEP and the wearing time of cochlear implant, but the N1 latency has no correlation to the wearing time of cochlear implant. These results are in concordance with Jeong et al. [31], finding significant negative correlation between the duration with the 1st implant and P1 latency, and longer cochlear implant use is associated with shorter P1 latency. Ponton et al. [32] found that the overall maturational sequence for P1 latency in implanted children was delayed by an interval approximating the period of auditory deprivation prior to implantation. This correspondence suggests that the “time in-sound,” which equals chronological age minus duration of deafness, determines the stage in the maturational process.

The CAP and SIR scores of the children who received the cochlear implant were positively correlated with the time of evaluation postimplantation and the wearing time of cochlear implant and have no correlation with the age of implantation, which suggested that the auditory performance and speech intelligibility of pediatric CI recipients were almost the same as those of the profound deafness children with early implantation. However, some studies have found that earlier implantation leads to better language comprehension outcomes, suggesting that auditory and speech functions may be influenced by the age of cochlear implantation and that there may be sensitive periods for central auditory and spoken language development at a younger age [33, 34], which is not inconsistent with our results.

Firszt et al. [22] found that variability in speech perception scores of adult cochlear implant recipients relates to neurophysiologic responses at different levels of the auditory pathway, including the CAEP. The CAP and SIR scores are commonly used to report hearing and speech outcomes after cochlear implantation. In our study, scores in both CAP and SIR test were significantly negatively correlated with the latency of the P1 of the electrically CAEP and have no correlation with the N1 latency which indicated that P1 latency may reflect the auditory performance and speech intelligibility of pediatric CI recipients.

However, in addition to the P1 and N1 components of the CAEP, the CAEP includes the P2 response. The latency of the CAEP P2 was significantly related to the speech perception scores [35]. Kelly et al. [36] found that P2 latency were associated with shorter durations of deafness and higher speech scores. P2 latency was found to be significantly shorter in adults with cochlear implants exhibiting “good” speech recognition performances as opposed to subjects exhibiting “poor” speech recognition performances [37].

All results together further confirm that neural encoding of the elicitation of electrophysiologic responses is related to speech perception. However, our results suggest that the latency of P1 could reflect the speech rehabilitation after cochlear implant. The role of the P2 component of the electrically CAEP in speech function was not to be studied because the P2 peak was not stably recorded, despite its prominence in both the infant and adult CAEP occurring at a peak latency around 140–210 ms in children 5–6 years [38].

The negative correlation between the latency of P1 and the scores of the CAP and SIR provides clinical evidence that the P1 latency could be regarded as a noninvasive biomarker to objectively assess outcomes of the auditory and speech function in deaf children fitted with cochlear implant. When speech discrimination is not unreliable to efficaciously evaluate aural rehabilitation in pediatric CI recipients, P1 might provide a clinical tool for assessing their efficacy and guiding postoperative intervention. However, interpretation of this study must be viewed with caution. It should be noted that the combined use of the P1 and other audiological test results can provide better guidance when clinicians make clinical decisions about the management of hearing-impaired children.

## Figures and Tables

**Table 1:** Demographic characteristics of the 43 children in this study.

Demographic characteristics (*n* = 43)	
Gender	Males: 30
	Females: 13
Deafness	Prelingual deafness: 43
	Postlingual deafness: 0
Age at implantation	Mean: 2.41 years
	Range: 0.79-6.42 years
Implantation side	Unilaterally right ear: 24
	Unilaterally left ear: 19
Wearing time of cochlear implant	Mean: 25.5 months
	Range: 2.0-120.3 months
Time of evaluation postimplantation	Mean: 4.56 years
	Range: 2.09-11 years

**Table 2:** The relation between the detection rate of P1 and N1 and implantation side and gender.

	*χ* ^2^	*P*
Detection of P1 and gender	3.022^a^	0.082
Detection of N1 and gender	0.341^a^	0.559
Detection of P1 and implantation side	0.096^a^	0.757
Detection of N1 and implantation side	0.094^a^	0.759

**Table 3:** The relation between the latency values of P1 and N1 and implantation side and gender.

	*t*	*P*
Detection of P1 and gender	0.693	0.493
Detection of N1 and gender	-0.459	0.650
Detection of P1 and implantation side	-1.020	0.315
Detection of N1 and implantation side	-1.057	0.301

## Data Availability

There is no data available for this study.

## Abbreviations

CAEP: Cortical auditory evoked potential
CI: Cochlear implantation
CAP: Categories of auditory performance
SIR: Speech intelligibility eating
SNHL: Sensorineural hearing loss
ANSD: Auditory neuropathy spectrum disorder
EALR: Evoked late latency response.
